# Influence of Dietary Biosynthesized Zinc Oxide Nanoparticles on Broiler Zinc Uptake, Bone Quality, and Antioxidative Status

**DOI:** 10.3390/ani13010115

**Published:** 2022-12-28

**Authors:** Hidayat Mohd Yusof, Nor’Aini Abdul Rahman, Rosfarizan Mohamad, Uswatun Hasanah Zaidan, Anjas Asmara Samsudin

**Affiliations:** 1 Department of Bioprocess Technology, Faculty of Biotechnology and Biomolecular Sciences, Universiti Putra Malaysia, Serdang 43400, Selangor, Malaysia; 2 Bioprocessing and Biomanufacturing Research Centre, Faculty of Biotechnology and Biomolecular Sciences, Universiti Putra Malaysia, Serdang 43400, Selangor, Malaysia; 3 Department of Biochemistry, Faculty of Biotechnology and Biomolecular Sciences, Universiti Putra Malaysia, Serdang 43400, Selangor, Malaysia; 4 Department of Animal Science, Faculty of Agriculture, Universiti Putra Malaysia, Serdang 43400, Selangor, Malaysia

**Keywords:** antioxidant, broiler, bone, feed supplement, nanobiotechnology, poultry, mineral, zinc, zinc oxide nanoparticles

## Abstract

**Simple Summary:**

Traditionally, inorganic zinc (Zn) was supplemented in broiler chicken diets at higher levels than recommended to meet optimal body requirements. Nonetheless, this practice poses challenges since increased Zn excretion in feces results in heavy metal contamination. The application of nanomaterial feed supplements in animal feed has recently evolved due to its unique properties in increasing nutrient utilization toward precision animal nutrition. Additionally, the high bioavailability and utilization rate of nanomaterial Zn are believed to improve broiler physiological function and production, and the results can support their practical application in the poultry industry. Therefore, this study aims to determine the effect of dietary biosynthesized zinc oxide nanoparticles (ZnO NPs) on broiler zinc uptake in serum and selected tissues, tibia bone quality parameters, and antioxidative status.

**Abstract:**

A total of 180 broiler chickens (Cobb500) were randomly allotted to five experimental groups consisting of six replicates and six birds in each pen. Each group was fed a basal diet supplemented with 100 mg/kg ZnO (control) and 10, 40, 70, and 100 mg/kg ZnO NPs for 35 days. Resultantly, Zn uptake and accumulation in serum, breast muscle, tibia bone, and liver were linearly and significantly (*p* < 0.05) increased with increasing dietary ZnO NPs supplementation at 100 mg/kg compared to the control group (dietary 100 mg/kg ZnO), implying effective absorption capacity of ZnO NPs. This was followed by lower Zn excretion in feces in broilers fed ZnO NPs compared to controls (*p* < 0.05). Furthermore, dietary ZnO NPs at 40, 70, and 100 mg/kg levels improved broiler tibia bone morphological traits, such as weight, length, and thickness. Similarly, tibia bone mineralization increased in broilers fed ZnO NPs at 100 mg/kg compared to the control (*p* < 0.05), as demonstrated by tibia ash, Zn, Ca, and P retention. Antioxidative status in serum and liver tissue was also increased in broilers fed dietary ZnO NPs at 70 and 100 mg/kg compared to the control (*p* < 0.05). In conclusion, dietary ZnO NPs increased Zn absorption in broiler chickens and had a positive influence on tibia bone development and antioxidative status in serum and liver tissue, with dietary ZnO NPs supplementation at 70 and 100 mg/kg showing the optimum effects.

## 1. Introduction

Zinc (Zn) is an essential trace element in poultry production. Zinc was reported as the most important restrictive trace element influencing growth performance among the four trace elements studied (copper, iron, manganese, and zinc) in broiler chickens [1]. This is due to the significance of Zn in a variety of physiological functions, including catalysis, structural support, regulatory support, and as an integral component of numerous enzyme structures and metalloenzymes [2]. Zn is also involved in the activity of a range of enzymes involved in carbohydrate, protein, and lipid metabolism, antioxidant defense system, and almost every metabolic pathway of the body. Additionally, Zn plays a vital role in hormone production and secretion, which contribute to growth, reproductive performance, immune function, and maintains normal feathers and bone formation [3]. Moreover, Zn is required in poultry for bone mineral homeostasis [4]. As a result, a severe Zn deficiency in broiler chickens could result in a range of abnormalities, including impaired decreased feed intake, poor growth, immune functions, disability of bones and joints, poor feathering, and death. Thus, it is necessary to supplement Zn in the broiler diet since the Zn level of plant-based feed is insufficient to meet body requirements. 

According to the National Research Council (NRC) [5], the recommended Zn requirement for broiler chickens is 40 mg/kg to support growth performance and production. On the other hand, the NRC’s recommended values for most trace minerals are based on earlier broiler strains and may be obsolete for the present commercial broiler strains. Traditionally, Zn in the broiler diet is supplied in the form of inorganic Zn, such as Zn oxide (ZnO) and Zn sulfate (ZnSO_4_) due to cost and commercial availability. However, the major downside of using inorganic Zn is its low bioavailability and retention due to its weak ionic bonds, which allow the metal ion to completely dissociate from the sulfate molecule once in contact with water [6], making it unavailable for absorption across the small intestine. Consequently, feed manufacturers use exceeded doses of Zn than the NRC’s recommendations ranging from 80 to 120 mg/kg [7,8], which is now a common practice to formulate diets with greater supplementary Zn to achieve optimal performance. The continuous use of high Zn supplementation results in increased excretion of undigested Zn in feces causing heavy metal contamination in the environment. Furthermore, high levels of Zn in the diet may affect the stability and digestibility of other trace elements including calcium (Ca), phosphorus (P), copper (Cu), iron (Fe), and cadmium (Cd), due to the binding activity of Zn ion with dietary components that are antagonistic to the former and restricting their absorption [9,10]. Hence, improving Zn bioavailability by using sources of Zn with better absorption and availability is a key element in resolving this problem. 

Nanotechnology has recently made tremendous development in a variety of scientific fields, including animal nutrition. Several studies have indicated that Zn in the form of nanoparticles (NPs) is an effective trace element for livestock and poultry [11,12,13,14,15,16]. The application of zinc oxide nanoparticles (ZnO NPs) demonstrated improvement in feeding efficiency and minimized the excretion of Zn into the environment. The novel properties of ZnO NPs include their extremely small particle size with high surface area and high chemical stability, which allows for excellent absorption efficiency and chemical reactivity while distinguishing them from their bulkier counterparts. In addition, ZnO NPs have a far better utilization rate due to direct penetration into the animal body and distribution into the blood and organs for prompt usage. The smaller particle size provides a unique delivery platform, allowing particles less than 5000 nm to pass through endocytotic and lymphatic membranes without interruption, and particles smaller than 500 nm to pass through cellular membranes [17,18].

Therefore, ZnO NPs are believed to have a higher bioavailability at higher or lower doses, making them ideal feed supplement candidates to replace conventional ZnO. Previous studies have indicated that supplementing nanosized Zn can improve absorption efficiency in various animal models in comparison to conventional Zn [13,19,20]. Furthermore, research has reflected that supplementing ZnO NPs in broiler chickens can improve body weight gain, feed conversion ratio, and meat quality, as well as the immune system and microbial population in the gastrointestinal tract (GIT) [11,12,21]. In addition, ZnO NPs have been used to modulate gut microflora, primarily to reduce pathogenic bacteria in broiler chickens, mice, and piglets [13,22,23,24,25,26,27]. Additionally, several studies have confirmed the potential antibacterial activities of ZnO NPs in vitro [28,29,30,31], allowing this unique material to be employed as an alternative to conventional antibiotics. Based on their promising characteristics, it is hypothesized that ZnO NPs exhibit positive impacts on broiler production. Nonetheless, research on the biological fate of ZnO NPs in the chicken GIT system in vitro and its relation to its efficacy in improving broiler Zn utilization in vivo is scarce. Therefore, the biological fate of biosynthesized ZnO NPs in the simulated physiological condition of the chicken GIT system and the effects of various levels of dietary biosynthesized ZnO NPs on broiler chicken Zn uptake in tissues, bone quality, mineralization, and antioxidative status were investigated in this study. To date, only a few studies have emphasized a safer and more sustainable synthesis of ZnO NPs for use as an animal feed supplement; hence, the present study advances green chemistry while ultimately contributing to precision animal nutrition.

## 2. Materials and Methods

### 2.1. Ethics Statement

All procedures used in this study were carried out in accordance with the guidelines approved by the Universiti Putra Malaysia (UPM) Institutional Animal Care and Use Committee (UPM/IACUC/AUP-R013/021).

### 2.2. Preparation and Characterization of Biosynthesized Zinc Oxide Nanoparticles (ZnO NPs)

The biosynthesized ZnO NPs powder was obtained and prepared following our prior research [32,33]. Briefly, a zinc-tolerant *Lactobacillus plantarum* TA4 was used to synthesize the ZnO NPs. The shape and size of ZnO NPs were determined using a high-resolution transmission electron microscope (HR-TEM) (JEM-2100F, Jeol, Tokyo, Japan) following our previously described procedure [28]. 

### 2.3. In Vitro Study of ZnO NPs Dissolution in the Simulated Physiological Condition of Poultry GIT System

The in vitro Zn dissolution of ZnO and ZnO NPs was determined under simulated physiological conditions of the poultry GIT system following the procedure described by Matin et al. [34] and Cho et al. [35] with some modifications. Table 1 presents the three parts of poultry GIT, as well as their pH values and average transit time. In brief, 2.0 g NaCl was dissolved in 1 L dH_2_O, and the pH was adjusted to 3.0, 6.2, and 5.8 (Table 1) using 2N HCl. For simulated gastric fluid, 3.2 g pepsin (Sigma-Aldrich, St. Louis, MO, USA) was dissolved; for simulated intestinal fluid, 87.5 mg bile salt and 25 mg pancreatin (Sigma-Aldrich, St. Louis, MO, USA) were dissolved. Amounts of 100 µg/mL of ZnO NPs were suspended in a prepared solution and incubated on a rotary shaker (100 rpm) at 37 °C. The incubation time was carried out in accordance with the GIT parts transit time shown in Table 1. After the incubation, ZnO NPs-free supernatants were collected by high-speed centrifugation (20,000× *g*) and the supernatant was then syringe filtered (0.2 µm pore). The dissolved Zn concentration was determined by inductively coupled plasma atomic emission spectroscopy (ICP-OES) (Optima 3700, Perkin Elmer, Waltham, MA, USA). The experiments were performed in triplicate.

### 2.4. Experimental Birds, Husbandry, and Diets

The experiment was conducted in the Poultry Unit, Faculty of Agriculture, Universiti Putra Malaysia, Malaysia. A total of 180 one-day-old broiler chicks (female; Cobb500 strain) were purchased from a local commercial producer, weighed, and randomly allotted into five dietary treatment groups (*n* = 36) with six replicates of six birds each (initial weight 41.1 ± 1.1 g). The chicks were raised in battery cages (90 cm width × 116 cm length × 50 cm height). The house temperature was set at 32 °C from day 1 until day 14 and reduced gradually until 25 °C was reached and maintained until day 35, on which no supportive heat was provided. The average relative humidity ranged between 60 and 75%, and the birds were provided with continuous fluorescent light following the lighting program outlined in the Cobb 500 Management Guide [36] throughout the experimental period. All of the birds were vaccinated against Newcastle and infectious bronchitis (ND-IB) disease on the 7th and 21st days via eye drops and against the infectious bursal disease (IBD) disease on the 14th day via drinking water. Birds were fed according to two phases of feeding periods: a starter diet (1 to 21 days) and a finisher (22 to 35 days). The dietary groups consisted of: a basal diet with 100 mg/kg ZnO (G1, control); basal diet + 10 mg/kg ZnO NPs (G2); basal diet + 40 mg/kg ZnO NPs (G3); basal diet + 70 mg/kg ZnO NPs (G4); and basal diet + 100 mg/kg ZnO NPs (G5). The basal diets formulated for each phase were based on corn–soybean meal following the nutrient requirements of Cobb500 broiler chickens (Table 2). The mineral premix was obtained from a commercial company (Peterlabs, Malaysia) for a customized premix formulation without the inclusion of Zn. The bulk ZnO and ZnO NPs were mixed prior to feed preparation in the treatment groups using a small-scale horizontal batch mixer (GEMCO Energy Machinery, Anyang, China). Feed in mash form and drinking water were provided *ad libitum* throughout the experiment for all experimental groups.

### 2.5. Slaughtering and Sample Collection

At the end of the experiment (day 35), 12 birds per treatment (2 birds per pen) were selected at random as representative samples, weighed individually, and slaughtered for sampling in accordance with Halal procedures, as defined in the Department of Standards Malaysia, 2009 protocols (MS1500:2009). The birds were slaughtered by a qualified technician by severing the jugular veins, carotid arteries, trachea, and esophagus with a razor-sharp knife in a slaughterhouse. About 15 mL of blood was collected from the jugular vein in a plastic serum tube during the slaughtering. The blood samples were allowed to clot before being centrifuged at 2000× *g* for 15 min at 4 °C to obtain the serum. The resultant serum was collected into tubes and stored at −80 °C until further analysis. The liver and breast muscle tissues were collected. A portion of liver tissue was sliced and frozen in liquid nitrogen and stored at −80 °C for antioxidant assay. Further, the sample preparation for Zn determination in liver and breast muscle samples was adapted from a previously described procedure by Qudsieh et al. [37] with some modifications. Briefly, the samples were prepared by mincing the meat and drying it at 105 °C for 48 h before being finely ground and stored for Zn concentration analysis.

The right and left tibia bones were removed between the tibial–tarsal joint and the tibial–femoral joint and boiled in deionized water for 5 min to remove all adhered soft tissues and cartilage caps before being dried at 105 °C for 24 h. The dried right tibia bones were used for determining the tibia traits including weight, proximal length, and lateral cortex thickness. The length and thickness were measured using digital calipers. Robusticity index (RI) (cm/g) was calculated by dividing the tibia proximal length (cm) by the dried tibia weight (g) [38]. Moreover, the left tibia bones were defatted by soaking in 100% petroleum ether for 5 h until fat residues were removed. The defatted bones were dried at 105 °C for 24 h to remove any excess ether. After drying, the dry-defatted left tibia was crushed and then ashed in a muffle furnace at 600 °C for 12 h, cooled in a desiccator, and weighed. Tibia ash percentage was calculated and expressed as a percentage of dry weight. The tibia ash was used to measure the mineral concentration analysis. 

Meanwhile, the excreta were collected for 4 d after the morning feeding on days 18, 19, 20, and 21 for the starter diet and on days 31, 33, 34, and 35 for the finisher diet. The samples were then pooled and prepared by carefully removing feed and feathers before being oven-dried at 105 °C for 48 h. Then the samples were ashed at 600 °C for 4 h and stored until Zn concentration analysis.

### 2.6. Acid Digestion and Mineral Determination

The Zn concentration analysis was performed on the liver, breast muscle, blood serum, tibia bone, and excreta. Meanwhile, the calcium (Ca) and phosphorus (P) concentration was performed on tibia bones. Briefly, approximately 1 g of the samples were digested using 10 mL 65% nitric acid and heated at 100 °C for 40 min on a hotplate until the solution turned clear. The cooled digested samples were then diluted with ultra-deionized water to the required volume for mineral analysis. Further, the serum Zn concentration was prepared using a method described by Laur et al. [39] with some modifications. Briefly, about 100 µL of serum were added to a 15 mL polypropylene tube and 200 µL of 65% HNO_3_ and 100 µL hydrogen peroxide (H_2_O_2_) were added. The mixtures were then vortexed and heated in a water bath at 60 °C for 90 min. The digested samples were then diluted with ultra-deionized water. The mineral concentration was determined by inductively coupled plasma emission spectroscopy (ICP-OES) (Optima 3700, Perkin Elmer, Waltham, MA, USA). An internal standard (Zn, Ca, and P) was used for calibration standard solutions. The values were adjusted according to the dilution factor. 

### 2.7. Evaluation of Antioxidative Status

The antioxidative status of the broiler chickens was evaluated by determining the serum and liver concentrations of malondialdehyde (MDA) and activities of superoxide dismutase (SOD), and catalase (CAT). Measurements were carried out using reagent kits from BioAssay Systems (Hayward, CA, USA) following the manufacturer’s instructions. Briefly, the liver tissues were homogenized with ice-cold phosphate-buffered saline (PBS) and centrifuged at 2000× *g* at 4 °C for 15 min. The homogenated supernatant was then collected for MDA and antioxidant enzymes assay. 

### 2.8. Statistical Analysis

Data from the present study were analyzed by using a one-way analysis of variance (ANOVA) followed by Tukey’s post hoc test to determine statistically significant differences among treatments. The data analyses and graphs were performed using GraphPad Prism version 7.0 (GraphPad Software, La Jolla, CA, USA). The results in the figures and tables were expressed as mean ± standard deviation (SD). *p* < 0.05 was considered statistically significant.

## 3. Results and Discussion

### 3.1. Biosynthesized ZnO NPs

**Figure 1:**
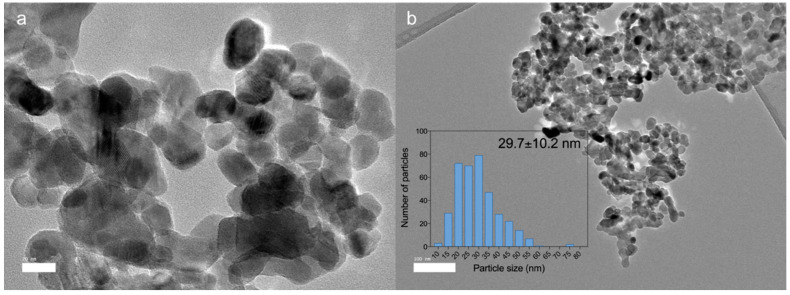
High-resolution transmission electron microscope (HRTEM) micrographs of biosynthesized ZnO NPs used in this study. (**a**) Micrograph of biosynthesized ZnO NPs at scale bar = 20 nm. (**b**) Micrograph of biosynthesized ZnO NPs at scale bar = 100 nm. The inset picture is the size distribution graph of biosynthesized ZnO NPs.

The production of ZnO NPs is fraught with challenges. ZnO NPs are conventionally synthesized by chemical and physical processes; however, these methods are not environmentally friendly. Thus, biological synthesis has been discovered to be an effective method due to its low cost and environmental friendliness [16]. In this study, we employed biosynthesized ZnO NPs that were previously produced using the zinc-tolerant probiotic *Lactobacillus plantarum* TA4 [33]. Figure 1 depicts an HRTEM image of biosynthesized ZnO NPs with an average particle size of 29.7 nm. The small particle size of ZnO NPs is expected to improve Zn absorption and bioavailability as a trace element in broiler chickens.

### 3.2. Dissolution of ZnO and Biosynthesized ZnO NPs under the Simulated Physiological Condition of the Chicken GIT System In Vitro

**Figure 2:**
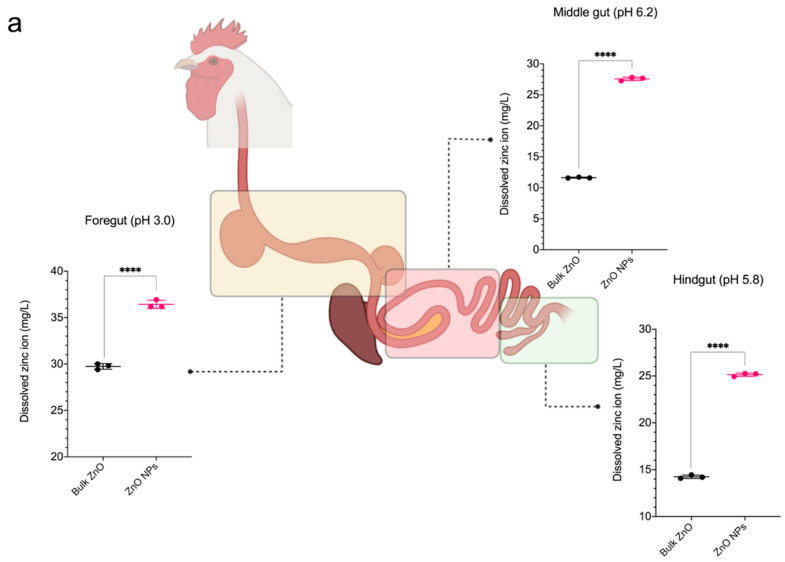

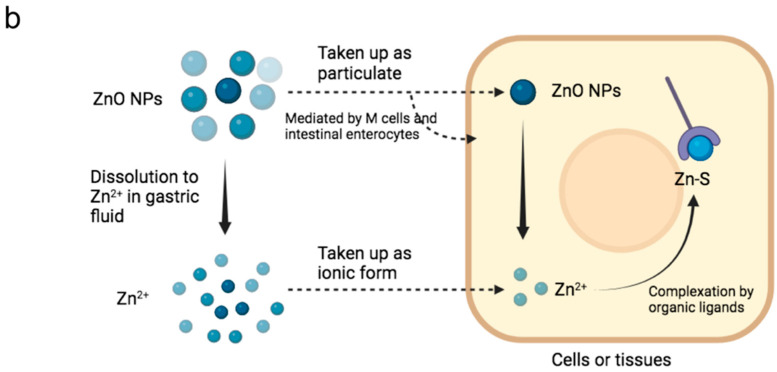
(**a**) Dissolution of ZnO and biosynthesized ZnO NPs under simulated physiological conditions of the chicken GIT system in vitro. (**b**) The biological fate of ZnO NPs and Zn^2+^ in cells or tissues is proposed. The low pH of gastric fluids, along with the presence of enzymes and organic compounds, may facilitate the dissolution of ZnO NPs. Cellular uptake of ZnO NPs can be either particulate or ionic; after uptake, the ZnO NPs dissolve as Zn^2+^ and interact with molecular or organic ligands, thus present in cells or tissues as ionic form. Data are expressed as mean ± SD. **** *p* < 0.001.

The biological fate of ZnO NPs in the GIT system is yet unknown, however, several studies suggest that ionic Zn is more accessible in tissues than particulate Zn [35]. Meanwhile, some studies advocated that Zn is absorbable in both forms [40,41]. Figure 2a depicts the dissolution of Zn^2+^ in vitro under a simulated physiological environment of a chicken GIT system. The data revealed that biosynthesized ZnO NPs dissociate more Zn^2+^ than bulk ZnO (*p* < 0.001) in both acidic and basic conditions. This indicated that ZnO NPs are more likely to dissolve under GIT conditions, potentially increasing the bioavailability of Zn in the form of ions and particles. Further, ZnO is considered insoluble in water but highly soluble in acidic solutions ranging from pH 2 to 4 owing to their inherent characteristics [42]. As a result, the dissolution of Zn^2+^ was observed to be greater in the acidic environment (foregut) for both bulk ZnO and ZnO NPs in comparison to other simulated GIT parts in the present study. These findings corroborate the results from several prior studies [35,42]. Cho et al. [35] revealed that ZnO NPs dissolve rapidly in acidic gastric fluid, implying that Zn^2+^ from ZnO NPs dissolve easily in the stomach before being absorbed and entering the systemic circulation. 

Figure 2b displays the proposed biological fate of ZnO NPs after they are ingested by chickens. The foregut of chickens, which includes the proventriculus and gizzard, has a low pH ranging from 2.5 to 3.5. In this context, when ZnO NPs are ingested, they mostly dissolve as Zn^2+^ in acidic gastric fluids and generate Zn cations that are taken up by the cells or tissues in an ionic form. Paek et al. [40] suggested that the main fate of ZnO NPs in tissues was the ionic form, as evidenced by the absence of particulate form on the liver and kidney tissue under transmission electron microscope (TEM) analysis. Similarly, Baek et al. [43] found no ZnO NPs particulate in TEM images of the liver and kidney of rats administered ZnO NPs but found significant Zn concentrations in organs tissues determined by ICP, confirming ZnO NPs ionized fate in the tissues. The researchers concluded that Zn^2+^ was taken up in tissues via interactions between Zn and sulfur-containing ligands in proteins, as evidenced by the detection of Zn-S peaks in rat organ samples using Fourier transform X-ray absorption spectroscopy (XAS) [43]. Nevertheless, ZnO NPs particulate uptake cannot be excluded since it can be mediated by normal intestinal enterocytes and M cells [44]. Several investigations [45,46,47] revealed cellular absorption of ZnO NPs as a particulate form, as evidenced by TEM images showing the presence of ZnO NPs in the tissue. Gilbert et al. [48] examined the uptake of ZnO NPs by human bronchial epithelial cells and discovered that ZnO NPs particulate in the cells as indicated by high-resolution X-ray spectromicroscopy and high elemental sensitivity X-ray microprobe analyses. Following uptake, ZnO NPs rapidly dissolved inside the cells, resulting in the formation of intracellular Zn^2+^ complexes with molecular or organic ligands [48]. Similarly, Jeon et al. [41] reported that ZnO NPs were absorbed into human intestinal cells in both particulate and ionic forms, and dissolved into ions over time. The researchers concluded that ZnO NPs were mostly transported as Zn^2+^, with a small amount being absorbed as particles. It is worth mentioning that ZnO NPs may be partially dissolved in biological fluids, resulting in the absorption of both particulate and ionic forms into the systemic circulation system, but they are predominantly localized in tissues as Zn^2+^ [40]. 

In this study, comparing the high dissolution of ZnO NPs to bulk ZnO suggests that the former provides high bioavailability of Zn in the body. The differences in the dissolution process between bulk ZnO and ZnO NPs can be explained by particle size differences. Bigger size particles have a low specific surface area, which results in a small contact area with the liquid medium, limiting chemical reactivity and delaying the dissolution process as compared to ZnO NPs [49]. Furthermore, ZnO NPs with smaller particle sizes contain higher proportions of surface atoms, which makes them more reactive [40]. The dissolution of Zn^2+^ in this study provides insight into the fate of ZnO NPs in simulated physiological GIT of chickens. Nonetheless, it does not represent the actual phenomenon in biological fluids due to the presence of various enzymes, organic and inorganic ligands, as well as feed components that may increase or decrease the dissolution rate. Thus, small particle size and fast dissolving ZnO source may overcome the gut’s absorbing capacity owing to their highly bioavailable, particularly in nutritional applications. Moreover, the rate of Zn absorption can be assessed by measuring Zn concentrations in serum and tissues, and the findings of this in vitro study will be used to support the biodistribution activity of ZnO NPs in comparison to ZnO in broiler chickens.

### 3.3. Zn Uptake and Concentration in Serum, Tibia Bone, Liver, and Breast Tissue 

To evaluate Zn utilization in broiler chickens, Zn status and uptake were observed in blood serum, different tissues, and their feces (Table 3). The concentrations of trace minerals in serum reflect mineral absorption homeostasis. The high dose of ZnO NPs supplementation in this study resulted in more Zn being delivered into the bloodstream. In comparison to G4 and G5, the serum Zn level of G2 and G3 was substantially lower (*p* < 0.05). However, no significant difference in serum Zn levels was detected between the control group (G1) and the G2 and G3, demonstrating that ZnO NPs enhance Zn bioavailability even at lower dosages than the bulk ZnO. It also demonstrates that the conventional ZnO employed in this study has a lower bioavailability. Blood serum Zn concentration can be used as an indicator of Zn bioavailability; a higher serum Zn content suggests that the Zn source is more bioavailable [26]. The increased serum Zn levels are probably due to the greater absorption of ZnO NPs given their extremely small particle size, which makes them easily absorbed by the villus epithelium and directly enter the bloodstream and distributed to targeted organs. Similarly, Milani et al. [26] reported that plasma Zn concentration in weaned pigs supplemented with 60 mg/kg ZnO NPs was statistically equal to those in controls receiving 3000 mg/kg ZnO, indicating that ZnO NPs had better absorption than their bulkier counterparts. Furthermore, Li et al. [50] found that supplementing ZnO NPs in weanling pigs increased serum Zn concentration to levels comparable to organic Zn, demonstrating a better Zn utilization rate in the body. It is also has been suggested that adequate or excessive Zn supplementation might significantly increase the concentration of metallothionein (MT) in the intestinal mucosa and influence the amount of Zn transported across the basolateral membrane into the blood circulation [51]. Notably, high Zn concentrations activate a variety of protective mechanisms, including MT induction. Inside the cell, MT functions as a Zn ion chaperone, mediating Zn transport and availability to other Zn-binding proteins [51]. 

The liver of broiler chicken is an essential organ that performs a variety of activities, including lipid, carbohydrate, protein, vitamin, and mineral metabolism, site for detoxification and elimination of waste substances from the feed [52]. In addition, the liver also serves as the primary storage site for vitamins and some minerals in the body, hence, Zn accumulation in the liver may be used as an index of mineral storage in the body to assess Zn distribution. In this study, the analysis of Zn content in the liver demonstrated that the Zn concentration increased proportionally as the inclusion level of ZnO NPs increased, with G5 having a significantly higher Zn concentration (*p* < 0.05) (Table 3). Although there was no significant difference in liver Zn concentration in G1 to G4 (*p* > 0.05), the Zn concentration of dietary ZnO NPs at 40 and 70 mg/kg (G3 and G4) was numerically greater than the control group (ZnO 100 mg/kg). These findings are in agreement with those of Zhao et al. [12] and Kumar et al. [14] who reported a higher concentration of Zn in the liver tissue of broiler chicken fed ZnO NPs compared with conventional Zn. Likewise, El-Bahr et al. [20] found that dietary ZnO NPs increased Zn content in both liver and brain tissues of Japanese quails when compared to the control, with liver tissue retaining a higher Zn concentration than brain tissue. This might be attributable to the ability of ZnO NPs to penetrate hepatic cells through the blood or interstitial space. These findings are consistent with high Zn concentrations in the blood serum of broilers given ZnO NPs, indicating that ZnO NPs are efficiently absorbed in the broiler’s body. As stated previously, the liver is susceptible to Zn supplementation due to its role as a Zn reservoir, and this study demonstrated that ZnO NPs enhanced bioavailability by increasing Zn liver concentration in birds fed ZnO NPs diets.

Similar patterns were found in tibia ash and breast tissue, where supplementing ZnO NPs at 100 mg/kg resulted in significantly increased Zn concentrations than other treatments (*p* < 0.05) (Table 3). Skeletal muscle and bone contain over 90% of total body Zn in humans, but just 55% in chickens, with skin and feathers accounting for about 31% of total body Zn [53]. Furthermore, body tissues that are high in Zn include bone, liver, kidney, pancreas, and Zn may redistribute across body tissue such as muscle, which is influenced by the level of Zn supplementation in animal diet [3]. As a result, measuring Zn retention in breast muscle can reflect Zn biodistribution throughout the broiler body. The present study reflected that birds fed ZnO NPs at 40, 70, and 100 mg/kg exhibited higher Zn content than ZnO, indicating that ZnO NPs are readily absorbed and delivered to muscle tissues. Bones have been evaluated as the most sensitive index for Zn bioavailability in broiler chickens, regardless of Zn supply or dosage, due to their function as a Zn reservoir in the body [54]. In this study, the tibia ash of broilers fed with ZnO demonstrated a lower Zn concentration when compared to broilers fed with ZnO NPs at low dosages (Table 3). Additionally, supplementing ZnO NPs at a 100 mg/kg diet retained more Zn in tibia ash than ZnO (*p* < 0.05), indicating higher Zn deposition in bone. Mohammadi et al. [55] also demonstrated that supplementing broiler chickens with ZnO NPs enhanced Zn concentration in the tibia ash more than the conventional ZnSO_4_. On the other hand, Kumar et al. [14] reported that supplementing broiler chicken with ZnO NPs at 27.5 mg/kg decreased Zn concentration in tibia ash. This inconsistency in Zn concentration might be attributed to a variety of factors, including health status, environmental conditions, the size of ZnO NPs used, and diet quality. In this study, it was evident that the Zn concentration in blood serum, tibia ash, breast, and liver tissue of broilers fed dietary ZnO NPs at 100 mg/kg was two-fold higher than that of broilers fed dietary ZnO at 100 mg/kg, indicating that ZnO NPs have a better absorption efficiency than their bulkier counterparts. 

### 3.4. Fecal Zn Excretion

Concerns with feeding broilers excessive Zn diets include the risk that they would excrete manure containing high levels of undigested Zn. This excess Zn fecal can pose a serious environmental risk by contaminating the water and soil with heavy metals, resulting in metal toxicity to plants and soil microbes. Table 3 depicts that ZnO supplementation (G1) resulted in a higher Zn concentration in excreta compared to other treatments (*p* < 0.05) on days 21 and 35. Furthermore, the concentration of Zn in excreta increased linearly as the amount of ZnO NPs supplied increased. Broilers fed high ZnO NPs (G5) exhibited significantly lower fecal Zn concentrations than the control group (G1) even at the same dosage (*p* < 0.05). Similarly, Kumar et al. [14] demonstrated that ZnSO_4_ supplemented broilers excreted more Zn in feces than ZnO NPs at 100 mg/kg concentration. Previous studies have also found that increasing Zn supplies increases fecal Zn excretion, regardless of Zn source, demonstrating a strong homeostatic control in Zn absorption [14,26,56].

The present study reflects that birds fed with ZnO excreted more Zn and demonstrated a poorer absorption of Zn in the body, thereby resulting in the release of more Zn into the environment. Low Zn excreta in broiler-fed ZnO NPs implies good absorption efficiency, as evidenced by serum, tibia, and tissue Zn content results in this study, which also elucidates how the birds utilize various levels of dietary Zn. Conventional inorganic Zn such as ZnSO_4_ tends to dissociate in the GIT of broiler chickens in a low and basic pH environment due to their electrovalent bonds [38] before interacting with other dietary components. As a result, Zn is released in the feces rather than absorbed across the small intestine [57]. Furthermore, inorganic Zn can bind with phytic acid, which is present in most broiler grain-based diets, and impairs Zn absorption and makes them less available [3,58]. On the other hand, organic Zn could not interact with phytic acid given that it lacks the free divalent cations required for chelation in the gut, hence it is metabolized in a variety of ways to facilitate absorption which makes it more bioavailable than its inorganic counterpart [3]. Burrel et al. [56] found that feeding organic Zn reduced feces Zn excretion compared to inorganic Zn as the former involved more absorption sites or transporters in the intestine, thereby increasing Zn retention. The high stability of organic Zn in the upper GIT also allows it to reach the small intestine where it is absorbed, which improves its bioavailability [57]. Nevertheless, employing organic Zn as a feed supplement is not economically effective, thus limiting its use. Resultantly, ZnO NPs at low or high doses might be employed as dietary strategies to replace overfeeding conventional Zn without jeopardizing animal health, performance, or the environment. 

### 3.5. Tibia Bone Traits, Ca, and P Retention

**Figure 3:**
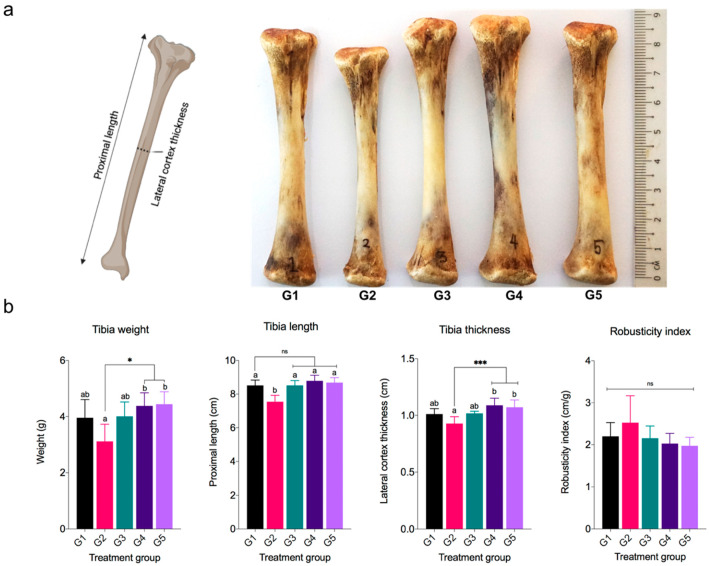

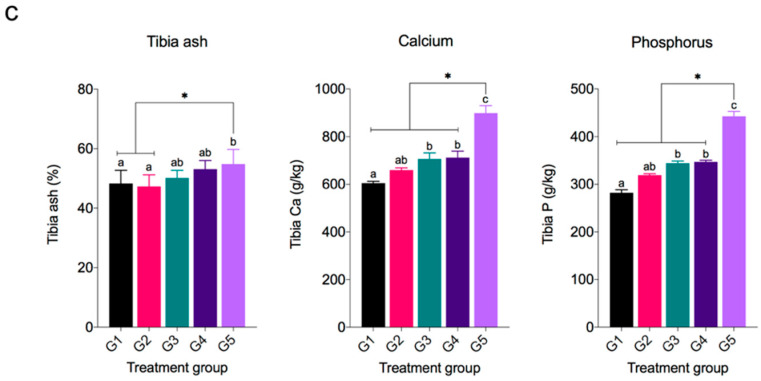
Tibia bone morphological characteristics and mineral concentration of broilers in response to the dietary treatments. (**a**) Morphological measurement and representative of dried tibia bone from each treatment group, (**b**) tibia bone morphological characteristics, and (**c**) tibia ash and mineral concentration (Ca and P). Data are expressed as mean ± SD. ^abc^ Values labeled with different superscripts differ significantly (*p* < 0.05). ns = not significant, * *p* < 0.05, *** *p* < 0.002.

The weight, length, thickness, and mineral content of bones have been studied as indicators of bone status and responsiveness to trace element supplementation. Bone is a vital organ that not only provides mobility, support, and protection to the body but also serves as a reservoir for essential minerals including calcium (Ca) and phosphorous (P) [9,59]. Skeletal disorders, particularly leg-related problems such as lameness and tibial dyschondroplasia, are one of the most common health issues in the poultry industry and pose a direct threat to poultry morbidity and mortality [60]. This skeletal disorder is attributed to the lack of trace minerals, such as Ca, P, and Zn in the diet. Moreover, trace mineral deficiency in bone may also be caused by an imbalance in mineral supplementation, which results in antagonistic interactions between minerals and reduces the absorption of other minerals (e.g., Ca and P) [59]. A balanced diet, particularly micro and macronutrients, is thus a key modifiable factor in bone development, health, and bone mass maintenance. Furthermore, tibia ash and mineral concentration are thought to be more sensitive measures of mineral utilization efficiency than growth performance metrics [61]. As depicted in Figure 3b, the weight and thickness of the tibia bone in G2 were significantly lower (*p* < 0.05) than in G4 and G5. Furthermore, the tibia length in G2 was significantly lower (*p* < 0.05) compared to the other treatment groups. Meanwhile, the tibia weight, length, and thickness were not significantly different (*p* > 0.05) among G1 (control), G3, G4, and G5 despite the higher numerical values in the latter two groups. These findings revealed that supplementing broilers with ZnO NPs at lower concentrations had a direct influence on tibia morphological characteristics. This finding was consistent with the tibia Zn content at a low ZnO NPs inclusion level of 10 mg/kg, which demonstrated lower Zn retention compared to other ZnO NPs treatments and increased in line with an increment in ZnO NPs level (Table 3). Although supplementation with ZnO NPs at a lower dose of 10 mg/kg resulted in decreased tibia bone weight and length, no skeletal problems were observed in G2, which may be due to sufficient Zn supply in the body. 

The present study suggests that bone mineralization increases when Zn levels in bone tissues increase. Zn is required for bone growth and strength, and it is found in alkaline phosphatase, collagenase, and aminoacyl tRNA synthetase. These events play a crucial role in the production of bone tissue [62]. In addition, Zn promotes bone regeneration and is required for normal skeletal development and bone homeostasis due to its vital role in protein and collagen synthesis [54]. Thus, insufficient dietary Zn may cause a decrease in bone collagen turnover, leading to a reduction in bone density [63], and most likely, leg deformities. A previous study found that turkeys fed a Zn-deficient diet exhibited reduced growth and had shorter long bones [64]. Furthermore, excessive Zn intake can reduce the bioavailability of other minerals, resulting in poor bone mineralization [59,65]. This is owing to the high dissociation of Zn ions which bind and antagonize a wide range of dietary components, including minerals, causing other mineral absorption to be hindered [9]. Since ZnO NPs demonstrated a high dissolution in the foregut under low pH conditions in this study, the antagonistic effects of ZnO NPs on other minerals need to be assessed. In this context, the Ca and P retention can reflect the changes in Ca and P homeostasis and could be utilized as an indication of the effects of different Zn levels on Ca and P absorption, utilization, and retention. Bone contains about 99% Ca and 85% P [59]. The mineralization of Ca and P in the bone increases tibia weight as these two minerals are key inorganic components of bone with significant effects on bone mineral content and density [59]. Ca and P are essential for leg bone strength and bone health, particularly in fast-growing broilers [38]. Many studies have documented that severe Ca or P deprivation can lead to impaired bone mineralization as measured by bone ash content. Studies by Hamdi et al. [66] and Onyango et al. [67] found that the low-calcium diet reflected the lowest bone weight and ash content of tibia in broiler chicken. 

The present study found that supplementing ZnO (G1) resulted in low Ca and P retention in the tibia, whereas supplementing dietary ZnO NPs increased Ca and P retention, with a significant increase in Ca and P at 100 mg/kg (G5) (*p* < 0.05) (Figure 3c). Similarly, the percentage of tibia ash in G5 was significantly higher than in G1 and G2 (*p* < 0.05). Increased bone ash indicates enhanced bone mineralization as a result of increased mineral utilization. Furthermore, the remarkable increase in tibia Ca and P in G5 may be due to Zn reaching the optimal level in promoting calcium and phosphate-regulating hormones [68] such as parathyroid hormone (PTH) and fibroblast growth factor 23 (FGF23) [69], which controls the level of Ca and P and stimulates bone formation. This finding further suggests that the high Zn^2+^ dissociation in ZnO NPs did not affect Ca and P absorption; hence, it was hypothesized that the majority of the ZnO NPs were absorbed by the cells or tissues in the form of particulates. In contrast to Stewart et al. [65], high Zn diet supplementation has an antagonistic impact on normal Ca and P deposition in the bone of young rats. However, these results cannot be compared to those from previous studies that used fairly extreme Zn levels since the level of ZnO NPs used in the present study appears to be moderate. Furthermore, no significant differences were detected in the robusticity index (RI) among groups (*p* > 0.05), with G5 and G2 having lower and higher numerical values, respectively. The robustness of the bone is characterized as RI, and it is correlated with bone mineral content and density, with lower RI indicating stronger bones [38]. The findings revealed that broilers supplemented with 100 mg/kg ZnO NPs had a stronger bone, which corresponded to their tibia ash content, Zn, Ca, and P concentration, suggesting a good influence of dietary ZnO NPs on bone development. Thus, supplementing ZnO NPs at doses of 100 mg/kg did not interfere with normal Ca and P absorption in the current study. Likewise, adding ZnO NPs at low doses did not affect bone mineralization or cause skeletal disorder. Interestingly, this study found that supplementing low ZnO NPs at 10 mg/kg had the same effect as supplementing 100 mg/kg ZnO in terms of tibia morphological characteristics, tibia ash, and tibia mineralization (Figure 3). 

### 3.6. Antioxidative Status 

Commercial poultry production is associated with a variety of factors that affect broiler chicken productivity, particularly in tropical climates where heat stress is a factor. This stress caused oxidative damage in broiler chickens, which hampered growth and health [70]. Zn is an important coenzyme of over 240 enzymes in birds that plays a key role in the production and activation of several antioxidant enzymes, notably SOD, which accounts for approximately 90% of its structure [12]. Superoxide dismutase (SOD) is the main antioxidant enzyme in cells that are involved in the scavenging of free radicals and reactive oxygen species (ROS) [12]. In addition, catalase (CAT) and glutathione peroxide (GPx) are also part of the body’s natural defensive mechanism against ROS. SOD enzyme converts the highly reactive superoxide radical to hydrogen peroxide (H_2_O_2_) and subsequently to water and oxygen (O_2_) by the CAT enzyme. Meanwhile, GPx is a catalyst that facilitates the reduction of a wide range of hydroperoxides (ROOH and H_2_O_2_) [70]. Moreover, Zn competes with iron and copper for binding to the cell membrane, thereby reducing the production of free radicals and functioning as a direct antioxidant [71]. Table 4 shows the effects of ZnO NPs supplementation on antioxidant enzyme activities and MDA concentrations in the serum and liver of broiler chicken. Serum and liver SOD activities were significantly higher in G3, G4, and G5 treatment groups compared to control and G2 (*p* < 0.05). Furthermore, serum CAT activity in G4 and G5 was substantially greater than in the other groups (*p* < 0.05). Meanwhile, CAT activity in the liver was much greater in G5, followed by G1 (*p* < 0.05) compared to other groups. Furthermore, there was no significant difference in CAT activity in serum and liver among G1, G2, and G3 (*p* > 0.05). The current study found that birds fed 70 and 100 mg/kg ZnO NPs had greater SOD and CAT activities in their blood and liver tissue than those fed 100 mg/kg ZnO. This revealed that antioxidant activity appeared to be related to dietary ZnO NPs levels and its bioavailability as corroborated by serum Zn concentration in G4 and G5, which was numerically greater, indicating high Zn bioavailability (Table 4). 

Malondialdehyde (MDA) is a useful biomarker for measuring lipid peroxidation and ROS-induced oxidative damage [70]. In this study, MDA levels in serum and liver tissue exhibited no significant differences between treatment groups (*p* > 0.05), however, G1 and G5 had numerically lower MDA levels than the other groups. The presence of antioxidant activity is evidenced by a decrease in MDA, which was confirmed by an increase in SOD and CAT in birds fed ZnO NPs. Thus, SOD and CAT enzymes participate in the cellular scavenging of free radicals, which protects the cells against toxicity and lipid peroxidation [70]. Aligning with the present findings, Hafez et al. [71] and Zhao et al. [12] found an increase in SOD and CAT enzyme activities in the serum of broiler chickens fed ZnO NPs than conventional ZnO. They also discovered that increased antioxidant activities were accompanied by a decrease in MDA level, indicating that ZnO NPs are effective at activating and promoting antioxidant enzymes. Furthermore, Abedini et al. [21] reported increased SOD enzyme activity in the plasma pancreas and liver tissues of laying hens fed ZnO NPs and Zn-methionine than those fed ZnO, suggesting that ZnO NPs had comparable bioavailability to organic Zn. 

Several mechanisms are involved in cell defense against free radicals. It has been proposed that metallothionein (MT), a cysteine-rich heavy metal-binding protein, assists in cell defense against oxidants and electrophiles [72]. Zn induces and promotes MT synthesis via metal regulatory transcription factor 1 (MTF-1) in response to higher levels of free Zn [72]. It has been shown to scavenge free hydroxyl and superoxide radicals in rabbit liver via a radical quenching mechanism involving cysteine sulfur atoms [73]. As a result, the same levels of MDA may be attributed to MT antioxidant activity, which is accompanied by their response to increased Zn bioavailability, resulting in comparable oxidative damage prevention. The present study suggests that ZnO NPs supplementation increases Zn bioavailability, promotes the antioxidant enzyme and MT synthesis, and thereby protects cells from free radicals and lowers the MDA levels. 

## 4. Conclusions

Nanotechnology is without a doubt one of the most significant technologies of the twenty-first century. Biosynthesized ZnO NPs have emerged as an alternative to chemical and physically synthesized ZnO NPs, promoting green chemistry and a more sustainable method of ZnO NPs synthesis. In comparison to conventional ZnO, ZnO NPs demonstrated a high dissolution rate in a simulated physiological condition of the chicken GIT system, showing their distinctive properties. In this present study, dietary supplementation with biosynthesized ZnO NPs improved Zn absorption in broiler chickens compared to ZnO, as evidenced by higher Zn concentrations in blood, tissues, and tibia bone. This was followed by a significant decrease in Zn excretion in the feces of birds fed ZnO NPs, indicating their high utilization rate. Tibia bone characteristics and mineralization improved in birds fed ZnO NPs, particularly at the 100 mg/kg dietary level, as compared to controls. Furthermore, the high bioavailability of ZnO NPs increased antioxidant activity in serum and liver tissue, which was accompanied by a decrease in MDA levels. In conclusion, it was evident that supplementing broiler chickens with ZnO NPs increased their Zn uptake and had positive influences, with dietary doses of 70 and 100 mg/kg showing the optimal results. Finally, given the potential benefits of biosynthesized ZnO NPs as a feed supplement in broiler chickens over their bulkier counterparts, the nanotechnology strategy might be exploited as a new tool for precision animal nutrition. Furthermore, more research is needed to investigate the mechanisms of ZnO NP absorption and their toxic effects at the cellular level, and supplementing ZnO NPs with phytase would be an interesting combination for future studies to increase mineral bioavailability in broiler chickens.

## Figures and Tables

**Table 1:** Duration of transit time and pH in different gastrointestinal tract (GIT) parts of broiler chickens.

GIT Parts	Segment	pH Value	Duration of Transit Time (min)
**Foregut**	Crop, proventriculus, and gizzard	3.0	90
**Middle-gut**	Small intestine	6.2	60
**Hindgut**	Caecum and colon	5.8	30

**Table 2:** Feed ingredients and nutrient composition of experimental diets.

Item	Starter (Day 1 to 21)	Finisher (Day 22 to 35)
Ingredient composition (%)		
Corn	52.5	58.5
Soybean meal (45% crude protein)	37.65	31.0
Wheat pollard	1.35	1.00
Palm oil (Refine)	5.00	6.00
Dicalcium phosphate ^1^	1.60	1.85
Calcium carbonate	0.60	0.35
Salt (NaCl)	0.30	0.30
DL-Methionine (99%)	0.25	0.25
L-Lysine (78.5% )	0.25	0.25
Mineral premix ^2^	0.15	0.15
Vitamin premix ^3^	0.10	0.10
Choline chloride	0.10	0.10
Toxin binder ^4^	0.15	0.15
Calculated nutrient composition (% DM, unless stated otherwise)		
Metabolizable energy (Kcal/kg)	3008	3167
Crude protein	22.6	20.09
Crude fat	7.57	8.004
Calcium	0.9	0.76
Available phosphorus	0.45	0.38
Methionine	0.5	0.43
Lysine	1.32	1.05
Threonine	0.919	0.783
Na	0.23	0.23
Analyzed Zn (mg/kg) ^5^	20.83	23.33

^1^ Dicalcium phosphate contained: Phosphorus, 18%; Calcium, 21%. ^2^ The Zn-free mineral premix provided per kilogram of diet: Manganese oxide, 35 mg; Ferrous sulfate, 35 mg; Copper sulfate, 60 mg; Cobalt carbonate, 5 mg; Potassium iodine, 0.6 mg; Selenium vanadate, 0.09 mg. ^3^ Vitamin premix provided per kilogram of diet: Vitamin A (retinyl acetate), 10.32 mg; Vitamin D3 (cholecalciferol), 0.25 mg; Vitamin E (DL-tocopheryl acetate), 90 mg; Vitamin K, 6 mg; Vitamin B12 (cobalamin), 0.07 mg; Vitamin B1 (thiamine), 7 mg; Vitamin B2 (riboflavin), 22 mg; Folic acid, 3 mg; Vitamin B7 (biotin), 0.04 mg; Vitamin B5 (pantothenic acid), 35 mg; Vitamin B3 (niacin), 120 mg; and Vitamin B6 (pyridoxine), 12 mg. ^4^ Toxin binder contains naturally hydrated sodium calcium aluminum silicates to reduce the exposure of feed to mycotoxin. ^5^ The Zn content was measured using ICP-OES. DM: dry matter.

**Table 3:** Zn concentrations in blood serum, tibia bone, feces, and different selected tissues of broiler chickens fed ZnO and ZnO NPs at different supplemental levels.

Items	Treatment Groups ^1^					SE ^2^	*p*-Value ^3^
	G1	G2	G3	G4	G5		
Serum (mg/L)	3.13 ± 0.36 ^a^	2.09 ± 0.23 ^a^	3.16 ± 0.05 ^a^	6.05 ± 0.65 ^b^	7.74 ± 1.43 ^b^	0.593	<0.0001
Liver (mg/kg DM)	612.5 ± 28.8 ^a^	400.0 ± 27.0 ^a^	479.2 ± 25.2 ^a^	935.8 ± 24.3 ^a^	1771.7 ± 577.1 ^b^	211.6	0.0004
Tibia (mg/kg DM)	479.2 ± 15.3 ^a^	551.7 ± 16.3 ^ab^	658.3 ± 38.3 ^b^	663.3 ± 64.9 ^b^	871.7 ± 118.2 ^c^	51.84	0.0002
Breast (mg/kg DM)	77.5 ± 9.0 ^abc^	45.8 ± 26.0 ^a^	90.8 ± 15.9 ^ac^	110.0 ± 27.8 ^c^	172.5 ± 15.0 ^d^	16.37	0.0002
Excreta (g/kg DM)							
Day 21	7.03 ± 0.04 ^a^	2.94 ± 0.34 ^d^	2.97 ± 0.11 ^d^	3.94 ± 0.15 ^c^	4.78 ± 0.24 ^b^	0.167	<0.0001
Day 35	7.08 ± 0.11 ^a^	2.71 ± 0.15 ^e^	3.77 ± 0.05 ^d^	4.14 ± 0.03 ^c^	4.57 ± 0.12 ^b^	0.083	<0.0001

^1^ Zn content (mg/kg diet): G1 (100 mg ZnO) (control); G2 (10 mg/kg ZnO NPs); G3 (40 mg/kg ZnO NPs); G4 (70 mg/kg ZnO NPs); G5 (100 mg/kg ZnO NPs). Data were expressed as mean ± SD. ^2,3^ Standard error of the mean (SE) and *p*-value for all treatments. ^a–e^ Means within the same row with no common superscripts differ significantly (*p* < 0.05). DM: dry matter.

**Table 4:** Effects of different levels of ZnO NPs on antioxidant enzyme activity and malondialdehyde concentration in broiler chickens.

Variable	Treatment Group ^1^					SE ^2^	*p*-Value ^3^
	G1	G2	G3	G4	G5		
Serum							
SOD ^4^ (U/mL)	1.30 ± 0.11 ^a^	1.63 ± 0.19 ^a^	2.65 ± 0.05 ^b^	2.65 ± 0.11 ^b^	2.57 ± 0.12 ^b^	0.102	<0.0001
CAT ^5^ (U/L)	5.12 ± 2.13 ^a^	3.91 ± 2.09 ^a^	7.64 ± 3.65 ^a^	13.90 ± 3.55 ^b^	17.33 ± 0.53 ^b^	2.162	0.0004
TBARS/MDA (µM)^6^	3.56 ± 0.29 ^a^	4.07 ± 0.23 ^a^	3.90 ± 0.36 ^a^	3.74 ± 0.30 ^a^	3.36 ± 0.05 ^a^	0.219	0.0801
Liver							
SOD ^4^ (U/mL)	1.33 ± 0.30 ^a^	1.22 ± 0.38 ^a^	2.38 ± 0.19 ^b^	2.21 ± 0.33 ^b^	2.28 ± 0.07 ^b^	0.225	0.0007
CAT ^5^ (U/L)	0.62 ± 0.17 ^a^	0.38 ± 0.10 ^a^	0.64 ± 0.08 ^a^	1.18 ± 0.29 ^b^	1.82 ± 0.31 ^c^	0.1728	<0.0001
TBARS/MDA (µM) ^6^	6.83 ± 0.22 ^a^	7.36 ± 0.02 ^a^	7.31 ± 0.50 ^a^	7.13 ± 0.08 ^a^	6.76 ± 0.05 ^a^	0.203	0.0609

^1^ Zn content (mg/kg diet): G1 (100 mg ZnO)(control); G2 (10 mg/kg ZnO NPs); G3 (40 mg/kg ZnO NPs); G4 (70 mg/kg ZnO NPs); G5 (100 mg/kg ZnO NPs). Data are expressed as mean ± SD. ^2,3^ Standard error of the mean (SE) and *p*-value for all treatments. ^4^ SOD, superoxide dismutase, one unit corresponds to the amount of enzyme needed to scavenge dismutation of the superoxide radical. ^5^ CAT, catalase, expressed as U/L (one unit is the amount of CAT that decomposes 1 µmole of H_2_O_2_ per min). ^6^ TBARS/MDA, Thiobarbituric acid reactive substance/malondialdehyde, TBARS is expressed as µM MDA equivalents. ^a–c^ Means within the same row with no common superscripts differ significantly (*p* < 0.05).

## Data Availability

Not applicable.
